# Evolution of RNA- and DNA-guided antivirus defense systems in prokaryotes and eukaryotes: common ancestry vs convergence

**DOI:** 10.1186/s13062-017-0177-2

**Published:** 2017-02-10

**Authors:** Eugene V. Koonin

**Affiliations:** National Center for Biotechnology Information, National Library of Medicine, Bethesda, MD 20894 USA

## Abstract

**Abstract:**

Complementarity between nucleic acid molecules is central to biological information transfer processes. Apart from the basal processes of replication, transcription and translation, complementarity is also employed by multiple defense and regulatory systems. All cellular life forms possess defense systems against viruses and mobile genetic elements, and in most of them some of the defense mechanisms involve small guide RNAs or DNAs that recognize parasite genomes and trigger their inactivation. The nucleic acid-guided defense systems include prokaryotic Argonaute (pAgo)-centered innate immunity and CRISPR-Cas adaptive immunity as well as diverse branches of RNA interference (RNAi) in eukaryotes. The archaeal pAgo machinery is the direct ancestor of eukaryotic RNAi that, however, acquired additional components, such as Dicer, and enormously diversified through multiple duplications. In contrast, eukaryotes lack any heritage of the CRISPR-Cas systems, conceivably, due to the cellular toxicity of some Cas proteins that would get activated as a result of operon disruption in eukaryotes. The adaptive immunity function in eukaryotes is taken over partly by the PIWI RNA branch of RNAi and partly by protein-based immunity. In this review, I briefly discuss the interplay between homology and analogy in the evolution of RNA- and DNA-guided immunity, and attempt to formulate some general evolutionary principles for this ancient class of defense systems.

**Reviewers:**

This article was reviewed by Mikhail Gelfand and Bojan Zagrovic.

## Background

Replication of digital information carriers, specifically nucleic acids, is the central, distinguishing feature of life [1–3]. Only with the onset of replication with sufficient fidelity to ensure information transmission across generations, evolution by natural selection and drift can take off [4, 5]. An intrinsic feature of even the simplest evolving replicator systems is the emergence and persistence of parasitic genetic elements [6–8]. Since that earliest stage of evolution, the entire subsequent history of life was a story of host-parasite coevolution, given that, in the long run, hosts cannot purge parasites, primarily because this would require a drop in the horizontal gene transfer rate that would be incompatible with evolutionary stability [8–11]. Strikingly, in today’s biosphere, the most abundant entities are not cells but viruses: the counts of virus particles exceed cell counts by one to two orders of magnitude in most environments [12–16]. An equally striking, complementary fact established by comparative genomics is that the genomes of many eukaryotes, particularly complex multicellular forms such as mammals or flowering plants, consist mostly of sequences derived from mobile genetic elements (MGE) [17, 18]. Given the inevitability of genetic parasites, evolution of defense systems by the cellular hosts and their diversification in the course of the perennial host-parasite arms race is one of the central aspects in the evolution of life.

**Fig. 1:**
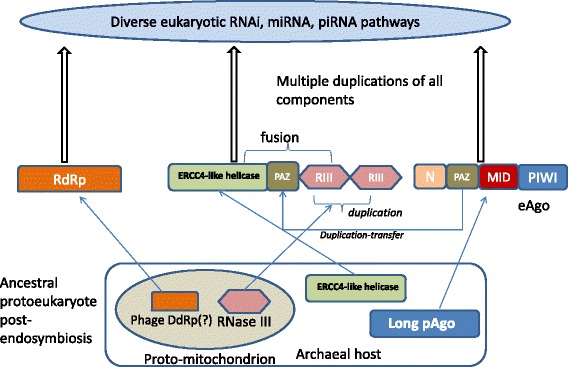
The evolutionary history of eukaryotic RNAi: assembly from diverse archaeal and bacterial ancestors. The “bacterial” and “archaeal” components of the RNAi protein machinery are assumed to have evolved from the proto-mitochondrial endosymbiont and its archaeal host, respectively. This scenario rests on the fact that RNase III is a protein that is nearly ubiquitous in bacteria but rare in archaea, and the (DNA-dependent) RNA polymerase that is thought to be the ancestor of the RNAi RdRp so far has been identified only in bacteriophages (not in archaeal viruses). However, it cannot be ruled out that these genes have been acquired by the mesophilic archaeal ancestor of eukaryotes (presumably, a member of the Lokiarchaeota) prior to endosymbiosis. RIII, RNAse III

The nucleic acid complementarity is the foundation of genomic replication, and hence a first principle of life. Thus, conceptually, it appears natural that an anti-parasite defense system would employ that same principle to recognize parasitic nucleic acids and target them for destruction by dedicated devices such as nucleases [19]. A defense system of this type would consist of a specificity component, a nucleic acid molecule of the optimal size for the recognition of a family of parasites, while avoiding self-recognition (hereinafter denoted the guide), and an operational (catalytic) component that is responsible for the efficient cleavage of the parasite genome (Fig. 1). In the extant defense systems, the catalytic function is allotted to dedicated protein enzymes but it stands to reason that in the primordial RNA world, the guide itself could be a ribozyme endowed with nuclease activity (Fig. 1).

The guide-dependent defense systems are indeed nearly ubiquitous among cellular organisms. In archaea and bacteria (hereinafter, collectively denoted prokaryotes), these include the recently discovered but common mechanisms of innate immunity centered around the prokaryotic Argonaute (pAgo) family nucleases [20] and the CRISPR-Cas systems which represent adaptive immunity [21–24]. Eukaryotes possess the enormously diversified network of RNA interference (RNAi) pathways, which include primarily innate immunity mechanisms, albeit in some cases, endowed with epigenetic immune memory (i.e. carry over of small interfering RNAs across generations), as well as a distinct type of adaptive immunity, the piwiRNA mechanism [19, 25–29]. Furthermore, in eukaryotes, the guide-dependent defense systems have expansively branched into mechanisms of gene expression regulation, and to a lesser extent, this trend is observed in prokaryotes as well.

Comparative genomic analysis has provided considerable insights into the origin and evolution of nucleic acid-guided defense systems. The relationships between prokaryotic and eukaryotic defense mechanisms are complicated and combine homology with functional analogy. In this article, without going in detail into the diversity of the eukaryotic RNAi systems, I present an overview of the evolutionary scenarios for the nucleic acid-guided defense and discuss the likely evolutionary forces behind the proliferation of the Ago-based mechanisms and the surprising demise of CRISPR-Cas in eukaryotes.

### The long journey of the Argonautes: direct inheritance of the prokaryotic guide-dependent innate immunity by eukaryotes

The Argonaute (AGO) genes were initially identified for their roles in plant development [30, 31]. The unusual name was coined because the AGO1 knockouts of *Arabisopsis thaliana* showed a peculiar leave shape, supposedly resembling a squid (Argonautes are not squids but a distinct, ancient branch of octopuses; the name seems to have been chosen for the sake of euphony) [30]. The subsequent developments around the Argonautes certainly beg changing the metaphor: this protein family has delivered the Golden Fleece. The first function of Ago characterized at the molecular level was the role of “slicer” in the eukaryotic siRNA response, i.e. the RNase that cleaves the target RNA base-paired with a small interfering (si)RNA [32–34]. Shortly thereafter, it has been established that enzymatically inactive members of the Ago family complexed with micro(mi)RNAs reversibly suppress the translation of the target mRNAs instead of cleaving them [35, 36].

The catalytically active moiety of the Ago proteins is the RNase H domain, one of the most common, versatile nucleases in cellular organisms and viruses that additionally adopted the ATPase activity in the nearly ubiquitous HSP70 family of molecular chaperones [37]. The distinct variety of the RNase H domain represented in Ago is known as the PIWI domain, after P element–Induced WImpy testis, a Drosophila mutant [38]. The RNase H domain encompasses the DED[DHK] tetrad of amino acid residues essential for catalysis which coordinate two divalent cations and catalyze RNA hydrolysis through a mechanism that is shared by a great variety of nucleic acid processing enzyme, not only nucleases but also polymerases.

Argonautes are large proteins of about 800–1200 amino acids that, in addition to the catalytic PIWI domain, contain non-catalytic domains, known as the PAZ (PIWI-Argonaute-Zwille), MID (Middle) and N domain, along with two domain linkers, L1 and L2 [20, 38–40] (Fig. 1). The MID domain is essential for binding the 5′-end of the guide and is present in all Ago proteins. The PAZ domain, which contains an OB-fold core typical of diverse nucleic acid-binding proteins, is not essential for the guide binding but stabilizes the guide from the 3’end. The N domain is not required for the guide loading but substantially contributes to the dissociation of the second, passenger strand of the loaded dsRNA and to the target cleavage. As discussed below, only the PIWI and MID domain are present throughout the Ago family whereas the PAZ and N domains are missing in some family members (Fig. 1).

Although initially Argonautes have been described as highly conserved eukaryote-specific proteins [30, 41], prokaryotic homologs of eukaryotic Ago (hereinafter, pAgo and eAgo, respectively) soon have been discovered in many bacteria and archaea. The spread of pAgo is limited, however, with about one third of the archaeal genomes and about 10% of the bacterial genomes shown to encode a member of this family [20]. The structures of several pAgo proteins have been solved, establishing the identities of the PIWI, PAZ and MID domain and unexpectedly demonstrating that at least some pAgos preferentially bind guide DNA rather than RNA molecules [42, 43]. These observations notwithstanding, the biological functions of pAgo have remained obscure. However, comparative analysis of the genomic neighborhoods of the pAgo genes has strongly suggested a role in defense [44]. Indeed, many of the pAgo genes are embedded in ‘defense islands’, the regions of bacterial and archaeal genomes that are significantly enriched for genes involved in various defense functions. Furthermore, even more tellingly, genes encoding pAgo variants with inactivated PIWI domains are often adjacent to genes encoding other nucleases, leading to the obvious hypothesis that these enzymatically inactive pAgos ensure the recognition of targets that are then cleaved by the associated active nucleases.

The hypothesis on the defense function of pAgo has been experimentally tested, with striking results, although the scope of the experiments remains limited. The ability to cleave target nucleic acids in vitro has been demonstrated for pAgos from the bacteria *Aquifex aeolicus* [42] and *Thermus thermophilus* [45], and the archaea *Methanocaldococcus jannaschii* [46] and *Pyrococcus furiosus* [47]. Notably, all three catalytically active pAgos employ ssDNA guides but differ in their ability to cleave RNA or DNA. In contrast, no nuclease activity has been demonstrated for the RNA-binding pAgo of the bacterium *Rhodobacter sphaeroides* that has been predicted to be inactive due to mutations in the catalytic center of the PIWI domain [48].

The defense functions have been demonstrated for the pAgo from *R. spheroides* [48] and *T. thermophilus* [49]. The *T. thermophilus* Ago restricts plasmid replication by cleaving the plasmid DNA using plasmid-derived small ssDNA guides. The mechanism of the guide generation is not understood in detail but it has been shown that the catalytic residues of the PIWI domain are required [49]. Accordingly, it appears likely that pAgo first shreds the plasmid DNA in a guide- (and presumably, sequence) independent manner and then becomes a target-specific nuclease after acquiring the guides. What determines the self/non-self discrimination at the first stage, remains unclear. For the *R. spheroides* pAgo, association with short RNAs that represent much of the bacterial transcriptome has been demonstrated [48]. In addition, this Ago is associated with ssDNA molecules complementary to the small RNAs, and this DNA population is enriched in “foreign” sequences, those from plasmids as well as mobile elements integrated into the bacterial chromosome. Apparently, in *R. sphaeroides,* pAgo samples degradation products of the bacterial transcriptome and then, via yet unknown mechanisms, preferentially generates complementary DNAs for foreign sequences that are used to repress the expression of the cognate elements. Whether or not the function of this catalytically inactive pAgo requires other nucleases, remains to be determined. Nevertheless, the presence of pAgo within evolutionarily conserved operons with genes for nucleases and helicases [20, 44] implies complex organization of the prokaryotic Ago-centered defense systems that remains to be investigated. Such experiments should clarify the mechanisms employed by the prokaryotic pAgo-centered defense systems to generate the guide RNA and DNA molecules and discriminate the genomes of parasites from those of the hosts.

Unlike the prokaryotic counterparts, the eukaryotic Ago-centered molecular machinery that is involved in RNAi has been studied in great detail. The diversity of the eukaryotic Ago family is staggering and involves multiple catalytically active (slicers) as well as even more numerous inactivated forms [50–53]. In addition to the defense function in the form of the small interfering (si) RNA branch of RNAi, eukaryotes possess a variety of regulatory pathways in the micro(mi)RNA branch [54–57]. Typically, the defense function of RNAi includes cleavage of foreign (virus) dsRNAs by active eAgo, whereas the miRNA pathways involve binding and reversible inactivation of mRNA, not involving cleavage, by inactive eAgo varieties (although in some cases, degradation of the mRNA by other nucleases is promoted). The antivirus and regulatory branches of RNAi appear to be directly linked: viral infection induces the formation of endogenous siRNA thst silence numerous host genes [58].

The structural and functional diversity as well as the details of evolution of eukaryotic RNAi are discussed in numerous reviews [59–61] and are not our primary concern here. Instead, we specifically focus on the prokaryotic roots of the eukaryotic RNAi (Fig. 1). In addition to eAgo, the second major protein that is involved in all RNAi pathways is Dicer which is responsible for the generation of siRNA from viral dsRNA and miRNA from precursor RNA molecules containing long double-stranded regions [62–65]. Similarly to eAgo, the Dicers form an extensive family of paralogs with distinct functions in various branches of RNAi [66–68]. Again, in parallel to Ago, Dicer is a multidomain protein that consists of a Superfamily II helicase, two RNase III domains and a Paz domain (Fig. 1) [69, 70]. Notably, in addition to its function in siRNA generation from viral dsRNA, Dicer has been shown to play a direct role in the defense against DNA viruses, such as adenoviruses, via cleavage of small RNAs that are involved in virus reproduction [71].

Apart from eAgo and Dicer, the third key protein of RNAi is a distinct RNA-dependent RNA polymerase (RdRp) that is involved in the amplification of the siRNA in most eukaryotes [72, 73]. The RdRp was lost at the onset of vertebrate evolution and in several other eukaryotic lineages but clearly is an ancestral component of eukaryotic RNAi [69]. These three proteins, eAgo, Dicer and RdRp, comprise the conserved core of RNAi (Fig. 1). The RISCs (RNA-Induced Silencing Complexes) include a variety of accessory proteins but these are not highly conserved in eukaryotic evolution [38]. Phylogenetic analysis of the Ago superfamily unequivocally places eAgo within a distinct branch of archaeal pAgo, namely the euryarchaeal branch. This specific origin of eAgo is notable in itself, given the recent identification of the archaeal group that is ancestral to eukaryotes, the Lokiarchaeota [74, 75]. The currently available Lokiarchaeum genome does not encode a pAgo homolog (as confirmed by BLASTP search of the Lokiarchaeum proteins using eAgo sequences as queries) suggesting that the actual archaeal ancestor of the eukaryotes acquired this gene from a euryarchaeal source, in agreement with the “mobile eukaryome” scenario [76]. Under this model, the genes that became eukaryotic signatures are frequently horizontally transferred in archaea such that the eukaryotic ancestor accumulated, more or less by chance, the entire “eukaryome”.

The apparent evolutionary history of Dicer is far more complicated than that of eAgo [69]. There is no ortholog of Dicer in either bacteria or archaea but the roots of individual domains are readily traceable (Fig. 1). RNase III is present in nearly all bacteria but only in very few mesophilic archaea that clearly acquired this gene via HGT [69, 77]. The helicase domain of Dicer comes from an altogether different line of descent: the closest homologs belong to the ERCC4 family of archaeal and eukaryotic helicases that are involved in DNA replication and repair (Fig. 1). Thus, the helicase moiety of Dicer is ultimately of euryarchaeal origin, possibly coming from the same source as eAgo. Finally, the PAZ domain is shared between Dicer and eAgo suggestive of an ancient recombination event between the genes encoding these key proteins of RNAi (Fig. 1). Finally, the distinct RdRp involved in RNAi adopts the double-psi beta barrel fold shared with the large subunits of DNA-dependent RNA polymerases (DdRp) and is most closely related to bacteriophage proteins that have not been characterized experimentally but are predicted to function as DdRp [78–80]. Thus, as indicated by the combined evolutionary evidence for its three key proteins, the eukaryotic RNAi system has a composite origin, with archaeal, bacterial and bacteriophages contributions (Fig. 1). It appears to have assembled from these components at an early stage of eukaryotic evolution, antedating the last common ancestor of the extant eukaryotes [69].

The RNAi is generally thought of as an innate immunity mechanism. However, there are two lines of evidence that link RNAi with adaptive immunity, blurring the boundaries between the two types of immunity. The first is epigenetic inheritance of siRNAs. It has been shown that at least in the nematode *Caenorhabditis elegans*, siRNA can be inherited across many generations, and moreover, that the duration of this inheritance is actively regulated [81–83]. The second mechanism with features of adaptive immunity is the piRNA branch of RNAi that is involved in transposon silencing in the animal germ line [84, 85]. The piRNAs are generated by processing of transcripts of degraded copies of transposons and loaded onto different Argonautes. The primary piRNAs are employed as guides to recognize and silence integrated transposons by triggering a modification in histone methylation that cause heterochromatinization. Notably, the piRNA pathway includes an additional regulatory loop, the so-called ping-pong mechanism in which the primary antisense piRNAs base pair with sense transcripts that are then cleaved by Ago to generate secondary, adaptive piRNAs [85].

### CRISPR-Cas: evolution of an adaptive immunity system from mobile genetic elements

The CRISPR-Cas systems became famous thanks to the enormous utility of some variants for genome editing and regulation [86, 87]. However, this form of immunity is also of immense fundamental biological interest, and moreover, its practical value is a direct consequence of the high specificity of the RNA-guided immunity mechanism [24]. The CRISPR-Cas is a bona fide adaptive (acquired) immunity system with a lasting memory of past infections stored in the form of unique spacers that are cut out of the target DNA and inserted between the repeats in a CRISPR array. The processed transcript of the spacer, the CRISPR (cr) RNA, is utilized as the guide RNA to recognize and cleave the target DNA or RNA. The size of the spacers, between 25 and 40 nucleotides, ensures extremely high specificity. The mechanisms of self/non-self discrimination and even the actual efficiency of these mechanisms in the case of CRISPR-Cas remain open problems [88]. The CRISPR-Cas systems have to discriminate between self and non-self sequences on two levels, namely adaptation (spacer selection) and target recognition. Obviously, recognition of the spacer itself by the guide crRNA has to be prevented for the CRISPR-Cas system to be functional. This is achieved via the Protospacer Adjacent Motif (PAM), a short nucleotide sequence that is required for the recognition of the cognate target sequence at both the adaptation and the interference stages but is absent from the CRISPR themselves [89, 90]. The problem of avoiding spacer acquisition from the host’s own DNA (and hence autoimmunity) is harder, and different CRISPR-Cas systems might solve (or ignore) it differently [91]. For some, strong preference for DNA that is actively replicated and subject to repair has been reported, thus biasing adaptation toward foreign DNA [92]. Others appear to be wasteful, with virtually no discrimination, resulting in extensive cell death that, however, is offset by survival of the few cells that adapt to the infectious agent [93].

The CRISPR-Cas systems demonstrate enormous diversity of gene composition, genomic loci organization and Cas protein sequences [23]. Nevertheless, extensive comparative analysis has revealed major evolutionary trends. These include multiple key contributions of mobile genetic elements; serial duplication of *cas* genes yielding functionally versatile effector complexes; and modular organization, with frequent recombination of the modules [23, 24, 94]. The two modules of the CRISPR-Cas systems include the suites of genes encoding, respectively, proteins involved in adaptation and effector functions, i.e. pre-crRNA processing, and target recognition and cleavage. Additionally, various proteins involved in ancillary roles such as regulation of the CRISPR response and probably CRISPR-associated programmed cell death, can be assigned to a third, accessory module.

The CRISPR systems are divided into two classes that differ with respect to the composition and complexity of the effector modules: multisubunit effector complexes in Class 1 and single, large effector proteins in Class 2 [23]. The adaptation module is more uniform across the diversity of the CRISPR-Cas systems and consists of the Cas1 and Cas2 proteins although in some CRISPR-Cas variants, additional proteins, such as the effectors themselves, e.g. Cas9, and accessory proteins, e.g. Cas4, are also required for adaptation [91]. Cas1 is the active integrase that catalyzes the protospacer excision from the target DNA and insertion into the CRISPR array whereas cas2 forms the structural scaffold of the adaptation complex [95, 96]. Comparative genomic analysis has revealed the likely ancestry of Cas1. Examination of the genomic context of *cas1* homologs that are not associated with CRISPR-*cas* loci led to the discovery of a novel superfamily of self-synthesizing transposons that have been denoted Casposons because the Cas1 protein they encode was predicted to function as the transposase (recombinase) [97, 98]. The integrase activity of the Casposon-encoded Cas1 subsequently has been validated experimentally [99], and similar target site specificities of Casposon integration and CRISPR spacer incorporation have been demonstrated [100]. Although the currently identified Casposons do not encode Cas2, some encode Cas4 and additional nucleases [98]. It seems likely that the entire adaptation module and perhaps even additional Cas proteins have been donated by a Casposon [101]. Furthermore, the prototype CRISPR repeats also could originate from the inverted terminal repeats of the ancestral Casposon. The ancestry of the effector module is less clear. Given that Class 1 CRISPR-Cas are almost universally present in archaea and also common in bacteria, whereas Class 2 systems are an order of magnitude less abundant, the multisubunit effector complexes of Class 1 are the most likely ancestral form [102]. Notably, despite the overall high diversity of the Cas proteins, the core subunits of the Class 1 effector complexes largely consist of multiple variants of the same domain, the RNA Recognition Motif (RRM) [94]. Some of the RRM domains possess nuclease activity whereas others are non-enzymatic RNA-binding proteins. This build-up of the effector complexes from ultimately homologous, even if highly diverged, building blocks implies evolution by gene duplication, with subsequent extensive diversification driven by the host-parasite arms race. Conceivably, the ultimate ancestor of the core Cas proteins could have been an RRM domain with a nuclease activity, such as that in the Cas10 protein, that gave rise to the extant multitude of active and inactivated versions. Subsequent evolution of the CRISPR-Cas systems also involved recruitment of additional proteins such as the helicase-nuclease Cas3 in the type I systems. What was the function of the original effector CRISPR-Cas module, before the fusion with the adaptation module, supposedly brought about by a Casposon? The previously proposed possibility is that the effector module evolved from an ancestral innate immunity system that acquired the adaptation capability following the integration of a Casposon next to the innate immunity locus [101]. So far, however, innate immunity systems homologous to CRISPR-Cas effector complexes have not been identified. Therefore, an alternative scenario would derive the Class 1 effector module from within the ancestral Casposon which, in this case, would be postulated to have encoded a RRM-domain nuclease.

The provenance of Class 2 effector modules is much clearer [102, 103]. The type II and type V effectors (Cas9 and Cas12, respectively) appear to derive from the abundant transposon genes known as *tnpB* which encode nucleases with the RNase H fold (also often denoted RuvC-like nucleases, after the homology to the bacterial Holiday junction resolvase RuvC). The role of TnpB in transposons remains unclear although it has been shown that this protein is not required for transposition [104]. In the Class 2 effectors, this nuclease cleaves the non-target DNA strand whereas the target strand (the strand complementary to the crRNA) is cleaved by an additional nuclease the identity of which differs between Cas9 and Cas12 [105, 106]. In the case of type CRISPR-Cas effector, Cas9, a distinct family of TnpB homologs, denoted IscB, has been identified as the direct ancestor as judged by high level of sequence similarity and the presence of a HNH inserted into the RuvC-like domain [107]. For the type V effectors, the direct ancestors are harder to identify but different subfamilies of TnpB appear to have given rise to different subtypes as indicated by sequence similarity and phylogenetic analysis [102, 103]. The type VI effectors, Cas13, are unrelated to those in other CRISPR-Cas types and contain two HEPN domains (Higher Eukaryotes and Prokaryotes Nucleotide-Binding, an acronym coined at a time when the actual activity of this domain was unknown) which cleave RNA targets [108, 109]. As with type V effectors, the exact ancestors of these proteins are difficult to pinpoint; either HEPN-domain containing Cas proteins of Class 1 CRISPR-Cas systems, such as Csx6 and Csn1, or HEPN-domain containing toxins could be implicated [103]. The most plausible evolutionary scenario is that Class 2 systems evolved when mobile elements encoding ancestors of Class 2 effectors integrated near orphan CRISPR arrays or displaced Class 1 effector operons. Type II, type V and type VI systems, and most likely, also different subtypes of type V apparently evolved independently on several occasions given their evolutionary affinity with different groups of TnpB or HEPN-containing proteins. Thus, the history of Class 2 systems involved the second, after the Casposons, major contribution of mobile elements to the evolution of the CRISPR-Cas adaptive immunity.

There are striking parallels between the designs and the likely evolutionary scenarios for the two best characterized adaptive immunity systems, CRISPR-Cas in prokaryotes and the protein-based adaptive immunity in jawed vertebrates [101]. Both systems function by rearranging the genome sequence, by inserting spacers into CRISPR arrays, in the case of CRISPR-Cas, and recombining immunoglobulin gene segments (VDJ recombination) in the case of the vertebrate immune system. Both these processes are mediated by recombinases derived from distinct, unrelated transposons, Cas1 in the case of CRISPR-Cas, and the Rag1-Rag2 recombinase in the vertebrate immune system. Conceivably, both systems evolved as a result of insertion of the respective, unrelated transposons next to an innate immunity locus. The recent discovery of a family of transposons that encode both Rag1, the catalytic subunit of the recombinase, and Rag2, the accessory subunit, reinforces this scenario [110]. A fundamental functional difference between the prokaryotic and animal adaptive immunity systems is that the adaptation in the former is inherited across generations whereas the vertebrate immunity only involves genome rearrangement in somatic cells. The other major difference is that, in the vertebrate immune system, target recognition involves protein-protein interaction as opposed to complementary interaction between nucleic acids.

### Convergent evolution of the two nucleic acid-guided defense systems of prokaryotes and their contrasting fates in eukaryotes

As outlined above, there are two (currently known) distinct nucleic acid-guided forms of defense in archaea and bacteria, the pAgo-centered innate immunity and the CRISPR-Cas adaptive immunity. These two classes of immune systems apparently evolved independently, largely from unrelated protein domains (with the caveat that the protein composition of the pAgo system is not known in detail) (Table 1). There seems to be, however, a degree of functional interaction between the two branches of the guided defense. As a case in point, a subfamily of pAgo genes are lodged within CRISPR-*cas* loci and catalyze RNA-guided cleavage of ssDNA, presumably in conjunction with CRISPR-Cas [111]. Conversely, it has been shown that expression of pAgo in the presence of the target plasmid stimulated also CRISPR-*cas* loci expression [112], suggesting that the innate and adaptive immune system in bacteria could be functionally coupled.

**Table 1:** The core proteins and domains comprising the RNA/DNA-guided immune systems^a^

pAgo: innate immunity in prokaryotes	Eukaryotic RNAi: innate immunity (piRNA branch: adaptive immunity)	CRISPR-Cas: adaptive immunity in prokaryotes
Adaptation/spacer acquisition		
NA	NA	Cas1: unique α-helical fold Cas2: RRM
Guide RNA processing/maturation and amplification		
pAgo: **RNase H, PAZ** additional nucleases(?)	Dicer: **ERCC4-like SF2 helicase** **RNase III** PAZ RdRp (double-psi beta barrel, DdRp homolog)	Class1: multi-RRM complexes Class2: [**RNase III**]; uncharacterized domains of effector proteins
Target recognition and cleavage		
pAgo: **RNase H, PAZ** additional nucleases(?)	eAgo: **RNase H, PAZ**	Class 1: **SF2 helicase,** HD nuclease, PolB-like/RRM nuclease Class 2: TnpB/RuvC (RNAse H fold), HEPN

^a^Only the key, evolutionarily conserved domains are included for each system. The domains that are homologous between different classes of RNA/DNA-guided systems are shown in bold type. For Class 2 CRISPR-Cas, RNase III is shown in brackets, to indicate that this is not a Cas protein

**Fig. 2:**
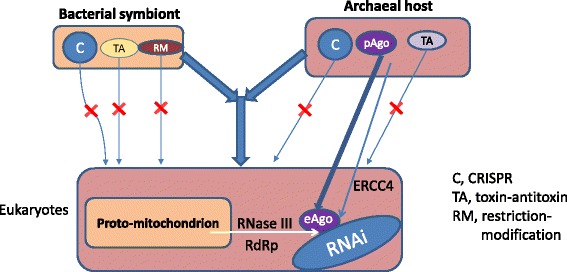
The fates of prokaryotic defense systems in eukaryotes. C, CRISPR-Cas; RM, restriction-modification; TA, toxins-antitoxins

The fates of the two major classes of prokaryotic nucleic acid-guided defense systems in eukaryotes could not have been more different. The pAgo system was directly inherited by the eukaryotes from the archaeal ancestor and extensively elaborated during the evolution of eukaryotes through the addition of extra components, such as Dicer and RdRp, and serial duplication (Fig. 2). The apparent assembly of the eukaryotic system from three distinct prokaryotic sources, namely the archaeal ancestry of eAgo and the helicase domain of Dicer, the bacterial ancestry of the RNase III domains of Dicer and the phage origin of the RdRp, emphasize the assignment of the origin of RNAi to the stage of eukaryogenesis [69]. At least under the symbiogenetic scenarios of eukaryogenesis, this stage of evolution is envisaged as a turbulent phase during which combination of genes of different origins including gene fusion were common and made diverse, substantial contributions to various functional systems of eukaryotes [113–116]. In addition to the dramatically increased complexity, the eukaryotic eAgo-centered RNAi machinery was reprogrammed to use RNA guides and to primarily target RNA. This major switch of specificity was apparently precipitated by the drastic change in the eukaryotic virosphere which is dominated by RNA viruses, in a sharp contrast with the DNA-dominated prokaryotic virome [117].

Unlike the pAgo-centered innate immunity, the CRISPR-Cas adaptive immunity was not inherited by eukaryotes. Strikingly, not only complete CRISPR-Cas systems but even individual Cas proteins have no eukaryotic homologs (apart from generic relationships among RRM domains, helicases and some nucleases). How can we explain this conspicuous absence of any traces of CRISPR-Cas in eukaryotes? One possibility is “frozen accident” whereby neither the archaeal host nor the bacterial endosymbiont that gave rise to mitochondria possessed CRISPR-Cas. Such a “frozen accident” cannot be ruled out because only a minority of bacteria carry CRISPR-Cas, and some mesophilic archaea, apparently including Loki, lack these systems as well [23].

However, there are also indications of biological causes of the exclusion of CRISPR-Cas from eukaryotes. CRISPR-Cas is not the only prokaryotic defense system that is missing in eukaryotes: also absent are RM and TA modules [118]. These defense systems share the key functional feature of requiring both a toxin (the active moiety) and an antitoxin, the regulatory moiety that prevents the toxic effect [119, 120]. The toxin and antitoxin have to be tightly co-regulated within the same operon, in order to efficiently control the toxic effect. In TA systems, the antitoxin directly interacts with the toxin, whereas in the RM systems, the modification component modifies the host DNA, making it resistant to the restriction component. Nevertheless, the general principle is the same for both these types of defense systems and involves essential coordination of expression and activity of the two components. It seems likely that this principle applies to CRISPR-Cas as well even though it is not a toxin-antitoxin module per se. Several Cas protein contain domains homologous to those in the common prokaryotic toxins including Cas2, which is a homolog of the VapD family interferases, and also HEPN domain-containing proteins (see above) [118]. Furthermore, toxicity has been demonstrated for the Csa5 protein although in this case, there are no homologs among known toxins [121]. Most strikingly, the recently characterized type VI CRISPR-Cas system appears to function as a toxin through the promiscuous RNase activity of its effector protein, Cas13a, which is induced by the recognition of the RNA target. Given that Cas13 proteins contain two HEPN domains, which is one of the signatures of prokaryotic toxins, type VI systems seem to present a clear-cut case of recruitment of toxins for functions in adaptive immunity. Although much more experimentation remains to be performed than had been done so far, taken together, all these findings appear compatible with the hypothesis on coupling between immunity and programmed cell death/dormancy by CRISPR-Cas systems [122–124]. Accordingly, the operon disruption ‘ratchet’ that was set into action by the emergence of eukaryotes destroyed the coupling and shifted the balance towards the toxic activity that would be incompatible with the survival of the eukaryotic cells [125]. Hence the rapid elimination of the RM, TA and CRISPR-Cas loci at the onset of eukaryotic evolution. Notably, however, the connection between RNAi and cell fate is likely to run deep in eukaryotes as well as indicated by the recent demonstration of the importance of RNAi for cell quiescence, the eukaryotic counterpart to prokaryotic dormancy [126].

## Conclusion

**Fig. 3:**
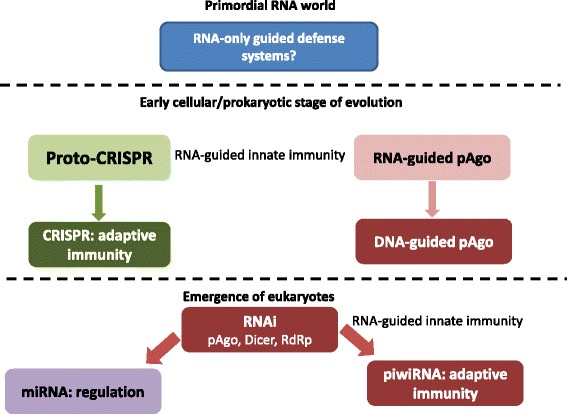
Evolution of RNA/DNA-guided defense and regulatory systems: from the RNA world to the present

The RNA/DNA-guided defense against genetic parasites is based on, arguably, the most fundamental chemical principle of life, nucleic acid complementarity, and might have been the first defense strategy to evolve, perhaps already in the primordial RNA world (Fig. 3). Archaea and bacteria possess two unrelated classes of guided defense systems, the Argonaute-based innate immunity and CRISPR-Cas adaptive immunity. The fates of these two defense systems in eukaryotes have been opposite: CRISPR-Cas was completely lost, whereas the Argonaute-based immune system underwent elaboration and enormous diversification. This proliferation of the Argonaute-based systems involved, in particular, the origin of the piRNA branch of RNAi that can be considered a distinct form of adaptive immunity. The striking contrast between the fortunes of the two systems could be due to the toxicity of certain Cas proteins that would be unleashed in eukaryotes because of operon disruption.

In eukaryotes, numerous inactivated Argonautes are recruited for regulatory roles, primarily in conjunction with miRNA, and the same can be expected to occur in prokaryotes although experimental data are currently lacking. The CRISPR-Cas system also assumed non-defense, regulatory functions in various bacteria even as these remain understudied as well [127]. The use of RNA or DNA guides for targeting unique areas of the genome (or transcriptome) is the most general possible strategy to achieve the goals of defense, attack and regulation.

Apart from the two defense systems discussed above, each of which also had been repeatedly recruited for regulatory functions, there are several other molecular machineries involved in natural genome engineering and regulation. A striking case in point is the system of DNA elimination during ciliate macronucleus development that relies on the so called scanRNAs and leads to the removal of varying fraction of the genome (up to more the 90%) in different ciliates [19, 128]. Many of the removed sequences originate from (largely inactive) transposable elements, and therefore, these mechanisms, in a sense, represent a distinct form of anti-parasite defense [129]. Although not studied in comparable detail, it appears most likely the DNA diminution that occurs during the developments of certain animals, e.g. Crustacea, employs analogous mechanisms [130]. A widespread regulatory system that functions on the RNA-guide principle is the prokaryotic small RNA regulation [131]. Bacterial and archaeal genomes encode from tens to hundreds small regulatory that in bacteria function mostly as complexes with the RNA chaperone Hfq [132]. This regulatory network is completely unrelated to either the Argonaute machinery or CRISPR-Cas. The existence of yet other RNA-guided pathways is indicated by the growing evidence of the major role of endogenous antisense RNA in the regulation of gene expression in eukaryotes [133, 134].

The universality of the central principle of RNA/DNA-guided defense and regulation strongly suggests that systems functioning on this principle evolved on multiple occasions in all forms of life. Even if the most common of these systems are already known, identification of new ones through combination of comparative genomics and experimentation is a major research challenge.

## Reviewers’ comments

### Reviewer 1: Mikhail Gelfand, Research and Training Center on Bioinformatics, A.A. Kharkevich Institute for Information Transmission Problems, Russian Academy of Sciences

This is a dual-purpose manuscript. Mainly it consists of a precise and complete, as of today, review of prokaryotic systems of nucleic acid (NA)-based immunity and prokaryotic origins of eukaryotic systems. However, I suspect that the real reason for writing this text has been the hypothesis explaining why adaptive NA-based CRISPR-Cas immunity has not been inherited by eukaryotes, unlike RNA-interference mechanisms.

Author’s response: *I appreciate the positive assessment and to a large degree concur with the reviewer regarding the incentive behind this manuscript. I would not go so far as to say that this is the “real” reason but, indeed, the main idea was to trace the dramatically different fates of different eukaryotic defense systems in eukaryotes, and in particular, to understand as best we can, why eukaryotes lack CRISPR-Cas.*

My marginal notes to the manuscript are mainly crossed-over. This is because in many places, where I had a suggestion or (so I thought) spotted an omission, this had been addressed on the next page. The author has not left out anything of importance; on the other hand, this style leads to a lot of minor stresses – a reader who has thought himself cleverer than the author is routinely disappointed. If this has not been the author’s intention, maybe it would be better to explain “epigenetic innate immunity” at the first occurrence (p.4, l.15-16), mention VDJ recombination at the first discussion of transposon contribution to adaptive immunity (p.11, l.15-18), etc. The author’s hypothesis – eukaryotes could not inherit systems that require tight co-regulation of components due to disruption of operons – looks interesting. However, there are a number of (admittedly minor) complications that need to be mentioned. Firstly, not all toxin-antitoxin (TA) systems in prokaryotes are encoded in the same operons; this is especially true for restriction-modification (RM) systems. The components may reside within one locus but in different operons, with correct regulation provided by a cis-encoded transcription factor, or even be distributed over the chromosome. (Here a possible explanation could be spatial separation of transcription and translation, slowing the response.) Secondly, and more generally, is tight co-regulation absolutely impossible in eukaryotes? (Here one might note that even if this is possible in modern eukaryotes, it might be difficult in early, primitive eukaryotic cells.) Thirdly, some eukaryotes have operons (likely of secondary origin, though). One of implications of the author’s theory is that other complexes or systems whose individual components may be poisonous or dangerous should (a) be encoded by operons in prokaryotes and (b) should be lost in eukaryotes. This may be testable, although requiring a lot of work. It looks like the situation will not be clear-cut. For instance, intermediate products of the riboflavin pathway are poisonous. In Firmicutes that enzymes forming the pathway are encoded by a single operon, often tightly regulated by a riboswitch. However, in Proteobacteria the genes are scattered, and often only one of then seems to be regulated (again, by a riboswitch); what is really surprising, this gene does not encode the enzyme responsible for the first reaction, but a middle one: hence, if the gene is repressed in conditions of abundant riboflavin, intermediates may still accumulate.

Author’s response: *the author’s intent certainly has not been to create “minor stresses” for the readers (even if one could argue that this might work as an attention getter). I considered the two specific suggestions made by the reviewer and indeed added a more concrete explanation of “epigenetic innate immunity”. As for mentioning VDJ recombination in the beginning of the discussion of the contribution of mobile elements to the evolution of defense systems, I do not really agree. I think in this case, a small element of suspense only helps, and the narrative comes to VDJ recombination exactly where it belongs. That said, the reviewer’s comment prompted me to slightly expand the discussion of the Rag1-Rag2 transposon and add a new reference. As for other possible “minor stresses” (etc), I am afraid I cannot easily identify those. Admittedly, this is likely to be an easier task for a reviewer/reader than for the author.*

*With regard to the exceptions from the co-regulation “rule” for toxin-encoding functional systems, I certainly appreciate these comments and expect them to be most helpful for readers. That said, this is what these cases are: (relatively) rare exceptions that emphasize the relevance of the main rule. Ditto for tight co-regulation in eukaryotes: it is not impossible but is much less common and much harder to achieve than it is in prokaryotes.*

*The prediction that other complexes or systems containing components that are dangerous in isolation should be tightly co-regulated (mostly, by virtue of operons) and likely lost in eukaryotes is pertinent and of major interest. I fully agree with the reviewer that this is testable albeit not easy. Such a project is underway, and hopefully, the findings that are likely to be generally compatible with the prediction will be published in a not so remote future.*

I do not agree with the author’s statement that nucleotide composition between plasmids and host chromosome may be used for self/non-self discrimination (p.6, l.45-47) – a protein (pAgo in this case) cannot measure the nucleotide composition of a chromosome – how would it collect statistical data? Moreover, nucleotide composition of the chromosome also is not uniform, given recently integrated mobile elements.

Author’s response: *I agree, this was a weak proposition. Dropped*.

The statement that miRNA pathways do not involve cleavage (p.7, l.2-29) seems to be too general: in mammals, miRNA binding yields mRNA degradation.

Author’s response: *This is about degradation by other nucleases not Argonaute. I included a comment to this effect.*

At p.8, l.54-55 does the author imply that same fold and same function equals homology?

Author’s response: *“Equals” might not be the right word here but the same fold does imply homology whereas the same function does not. This is not the place for a general discussion of this issue but the specific case of the RNA polymerase is, I believe, appropriately addressed in the cited references*.

### Reviewer 2: Bojan Zagrovic, Max F. Perutz Laboratories (MFPL), Department of Structural and Computational Biology, University of Vienna

The author presents a detailed, compelling and eloquent chain of arguments concerning the evolution of DNA- and RNA-guided immunity and, in particular, the evolutionary connections between the prokaryotic pAgo-centered innate immunity and CRISPR-Cas adaptive immunity on the one hand and eukaryotic RNAi and its diverse variants on the other. I find the discussion of the apparent lack of any CRISPR-Cas-related mechanisms in eukaryotes as a particularly novel and exciting contribution, which is likely to stimulate future discussion and work.

Author’s response: *I appreciate these positive comments*.

1. In an intriguing albeit speculative segment of the text (P12-13), the author draws a parallel between the organization and evolutionary developments of the adaptive immunity strategies in prokaryotes and jawed vertebrates. It would be interesting if the author could extend and strengthen the line of argument presented in this context. In particular, it may be interesting to explore the differences and the similarities between the two in relation to the fact that in the former the recognition of foreign elements occurs intra-cellularly, while in the latter it occurs extra-cellularly. Perhaps the fact that the former system is nucleic-acid-based and the latter protein-based may be related in part to this very fact.

Author’s response: *This is a perfectly salient and interesting point. Given the Biology Direct format, I believe that the comment will suffice to bring it to the readers’ attention*.

2. The author argues that the nucleic-acid complementarity is the most fundamental physico-chemical principle of life and that an RNA/DNA-guided defense system based on it could have evolved already in the RNA world context. Considering the recent results pointing at a possibility of co-evolution and a high level of intertwining of nucleic-acid and protein-based systems right from the very beginnings of life (see, for example, the results of Sutherland et al. who showed that chemical precursors of a number of nucleobases and amino acids can be obtained via prebiotic synthetic routes [135]) or the results demonstrating the possibility of complementary, specific interactions between nucleic acids and proteins (e.g. [136], it may be interesting to consider whether there are (were) any similar adaptive immunity defense systems that are (were) based on the direct recognition of nucleic acids and proteins through direct, non-covalent interactions. In other words, such recognition can also be thought of as a potentially evolutionarily old physico-chemical principle behind life and it would be interesting to consider whether it has ever been utilized for the purposes of differentiating between self and non-self in the context of a separate defense system. While there exist protein antibodies against nucleic acids (which are especially important in the context of autoimmune diseases), it is intriguing that the present day systems involve predominantly either nucleic-acid/nucleic-acid or protein/protein recognition.

Author’s response: *Proteins and more so peptides indeed could have been important components of (pre)biological systems from the earliest stages of their evolution. However, these must have been abiogenic or at least not nucleic acid-encoded peptide as discussed at length in an earlier paper* [137]*. Thus, it is difficult to imagine that these molecules contributed to self vs non-self discrimination at the earliest stages of evolution, whereas nucleic acids (most likely, RNA) appear to be well suited for this role since the very emergence of replication. As for the extant defense systems, specific recognition of nucleic acids by proteins is indeed widely utilized as demonstrated by the RM mechanism.*

1. In the title of the manuscript, it may be good to reverse the positions of “RNA” and “DNA”, considering the fact that the eukaryotes, which are named second, preferentially used the RNA-based systems.

Author’s response: *upon considering this suggestion, I decided to stick with the original title given that overall, RNA guides are much more common than DNA ones*.

2. P3L26 should be “particles”. 3. P5L26 “the Ago proteins” should be removed. 4. P8L12 it would be good to explain what RISC stands for. 5. P16L41 “RNA” missing?

Author’s response: *This is appreciated. Points 2–4 are taken care of but I am unsure about point 5 because there is no line 41 on p. 16*.

## Abbreviations

CRISPR-Cas: Clustered Regularly Interspaced Palindromic Repeats-CRISPR-ASsociated proteins
eAgo: Eukaryotic Argonaute protein
HEPN: Higher eukaryote-prokaryote nucleotide-binding domain
pAgo: Prokaryotic Argonaute protein
RM: Restriction-modification
RNAi: RNA interference
TA: toxin-antitoxin

## References

[1] Schroedinger E. What Is Life? with “Mind and Matter” and “Autobiographical Sketches”. Cambridge: Cambridge University Press; 2012.

[2] Crick F (1970). Central dogma of molecular biology. Nature.

[3] Koonin EV (2015). Why the Central Dogma: on the nature of the great biological exclusion principle. Biol Direct.

[4] Eigen M (1971). Selforganization of matter and the evolution of biological macromolecules. Naturwissenschaften.

[5] Koonin EV (2011). The Logic of Chance: The Nature and Origin of Biological Evolution.

[6] Smith JM (1979). Hypercycles and the origin of life. Nature.

[7] Szathmary E, Maynard Smith J (1997). From replicators to reproducers: the first major transitions leading to life. J Theor Biol.

[8] Iranzo J, Puigbo P, Lobkovsky AE, Wolf YI, Koonin EV. Inevitability of genetic parasites. Genome Biol Evol 2016, in press. 10.1093/gbe/evw193 PMC5631039 27503291

[9] Forterre P, Prangishvili D (2009). The great billion-year war between ribosome- and capsid-encoding organisms (cells and viruses) as the major source of evolutionary novelties. Ann N Y Acad Sci.

[10] Forterre P, Prangishvili D (2013). The major role of viruses in cellular evolution: facts and hypotheses. Curr Opin Virol.

[11] Koonin EV, Dolja VV (2013). A virocentric perspective on the evolution of life. Curr Opin Virol.

[12] Edwards RA, Rohwer F (2005). Viral metagenomics. Nat Rev Microbiol.

[13] Suttle CA (2005). Viruses in the sea. Nature.

[14] Suttle CA (2007). Marine viruses--major players in the global ecosystem. Nat Rev Microbiol.

[15] Rohwer F (2003). Global phage diversity. Cell.

[16] Rohwer F, Thurber RV (2009). Viruses manipulate the marine environment. Nature.

[17] Kazazian HH (2004). Mobile elements: drivers of genome evolution. Science.

[18] Cordaux R, Batzer MA (2009). The impact of retrotransposons on human genome evolution. Nat Rev Genet.

[19] Sabin LR, Delas MJ, Hannon GJ (2013). Dogma derailed: the many influences of RNA on the genome. Mol Cell.

[20] Swarts DC, Makarova K, Wang Y, Nakanishi K, Ketting RF, Koonin EV, Patel DJ, van der Oost J (2014). The evolutionary journey of Argonaute proteins. Nat Struct Mol Biol.

[21] Barrangou R, Marraffini LA (2014). CRISPR-Cas systems: Prokaryotes upgrade to adaptive immunity. Mol Cell.

[22] Marraffini LA (2015). CRISPR-Cas immunity in prokaryotes. Nature.

[23] Makarova KS, Wolf YI, Alkhnbashi OS, Costa F, Shah SA, Saunders SJ, Barrangou R, Brouns SJ, Charpentier E, Haft DH (2015). An updated evolutionary classification of CRISPR-Cas systems. Nat Rev Microbiol.

[24] Mohanraju P, Makarova KS, Zetsche B, Zhang F, Koonin EV, van der Oost J (2016). Diverse evolutionary roots and mechanistic variations of the CRISPR-Cas systems. Science.

[25] Zhou R, Rana TM (2013). RNA-based mechanisms regulating host-virus interactions. Immunol Rev.

[26] Szittya G, Burgyan J (2013). RNA interference-mediated intrinsic antiviral immunity in plants. Curr Top Microbiol Immunol.

[27] Wutz A (2011). RNA-mediated silencing mechanisms in mammalian cells. Prog Mol Biol Transl Sci.

[28] Holoch D, Moazed D (2015). RNA-mediated epigenetic regulation of gene expression. Nat Rev Genet.

[29] Gammon DB, Mello CC (2015). RNA interference-mediated antiviral defense in insects. Curr Opin Insect Sci.

[30] Bohmert K, Camus I, Bellini C, Bouchez D, Caboche M, Benning C (1998). AGO1 defines a novel locus of Arabidopsis controlling leaf development. EMBO J.

[31] Kidner CA, Martienssen RA (2004). Spatially restricted microRNA directs leaf polarity through ARGONAUTE1. Nature.

[32] Song JJ, Smith SK, Hannon GJ, Joshua-Tor L (2004). Crystal structure of Argonaute and its implications for RISC slicer activity. Science.

[33] Liu J, Carmell MA, Rivas FV, Marsden CG, Thomson JM, Song JJ, Hammond SM, Joshua-Tor L, Hannon GJ (2004). Argonaute2 is the catalytic engine of mammalian RNAi. Science.

[34] Lingel A, Izaurralde E (2004). RNAi: finding the elusive endonuclease. RNA.

[35] Hutvagner G, Zamore PD (2002). A microRNA in a multiple-turnover RNAi enzyme complex. Science.

[36] Nelson P, Kiriakidou M, Sharma A, Maniataki E, Mourelatos Z (2003). The microRNA world: small is mighty. Trends Biochem Sci.

[37] Majorek KA, Dunin-Horkawicz S, Steczkiewicz K, Muszewska A, Nowotny M, Ginalski K, Bujnicki JM (2014). The RNase H-like superfamily: new members, comparative structural analysis and evolutionary classification. Nucleic Acids Res.

[38] Cerutti L, Mian N, Bateman A (2000). Domains in gene silencing and cell differentiation proteins: the novel PAZ domain and redefinition of the Piwi domain. Trends Biochem Sci.

[39] Parker JS, Roe SM, Barford D (2005). Structural insights into mRNA recognition from a PIWI domain-siRNA guide complex. Nature.

[40] Parker JS, Barford D (2006). Argonaute: A scaffold for the function of short regulatory RNAs. Trends Biochem Sci.

[41] Tabara H, Sarkissian M, Kelly WG, Fleenor J, Grishok A, Timmons L, Fire A, Mello CC (1999). The rde-1 gene, RNA interference, and transposon silencing in C. elegans. Cell.

[42] Yuan YR, Pei Y, Ma JB, Kuryavyi V, Zhadina M, Meister G, Chen HY, Dauter Z, Tuschl T, Patel DJ (2005). Crystal structure of A. aeolicus argonaute, a site-specific DNA-guided endoribonuclease, provides insights into RISC-mediated mRNA cleavage. Mol Cell.

[43] Wang Y, Juranek S, Li H, Sheng G, Tuschl T, Patel DJ (2008). Structure of an argonaute silencing complex with a seed-containing guide DNA and target RNA duplex. Nature.

[44] Makarova KS, Wolf YI, van der Oost J, Koonin EV (2009). Prokaryotic homologs of Argonaute proteins are predicted to function as key components of a novel system of defense against mobile genetic elements. Biol Direct.

[45] Sheng G, Zhao H, Wang J, Rao Y, Tian W, Swarts DC, van der Oost J, Patel DJ, Wang Y (2014). Structure-based cleavage mechanism of Thermus thermophilus Argonaute DNA guide strand-mediated DNA target cleavage. Proc Natl Acad Sci U S A.

[46] Willkomm S, Zander A, Grohmann D, Restle T (2016). Mechanistic Insights into Archaeal and Human Argonaute Substrate Binding and Cleavage Properties. PLoS ONE.

[47] Swarts DC, Hegge JW, Hinojo I, Shiimori M, Ellis MA, Dumrongkulraksa J, Terns RM, Terns MP, van der Oost J (2015). Argonaute of the archaeon Pyrococcus furiosus is a DNA-guided nuclease that targets cognate DNA. Nucleic Acids Res.

[48] Olovnikov I, Chan K, Sachidanandam R, Newman DK, Aravin AA (2013). Bacterial argonaute samples the transcriptome to identify foreign DNA. Mol Cell.

[49] Swarts DC, Jore MM, Westra ER, Zhu Y, Janssen JH, Snijders AP, Wang Y, Patel DJ, Berenguer J, Brouns SJ (2014). DNA-guided DNA interference by a prokaryotic Argonaute. Nature.

[50] Hutvagner G, Simard MJ (2008). Argonaute proteins: key players in RNA silencing. Nat Rev Mol Cell Biol.

[51] Hock J, Meister G (2008). The Argonaute protein family. Genome Biol.

[52] Meister G (2013). Argonaute proteins: functional insights and emerging roles. Nat Rev Genet.

[53] Azlan A, Dzaki N, Azzam G (2016). Argonaute: The executor of small RNA function. J Genet Genomics.

[54] Iwakawa HO, Tomari Y (2015). The Functions of MicroRNAs: mRNA Decay and Translational Repression. Trends Cell Biol.

[55] Jonas S, Izaurralde E (2015). Towards a molecular understanding of microRNA-mediated gene silencing. Nat Rev Genet.

[56] Roberts TC (2015). The microRNA Machinery. Adv Exp Med Biol.

[57] Reis RS. The entangled history of animal and plant microRNAs. Funct Integr Genomics 2016. doi:10.1007/s10142-016-0513-0. 10.1007/s10142-016-0513-0 27549410

[58] Cao M, Du P, Wang X, Yu YQ, Qiu YH, Li W, Gal-On A, Zhou C, Li Y, Ding SW (2014). Virus infection triggers widespread silencing of host genes by a distinct class of endogenous siRNAs in Arabidopsis. Proc Natl Acad Sci U S A.

[59] Ding SW (2010). RNA-based antiviral immunity. Nat Rev Immunol.

[60] Wilson RC, Doudna JA (2013). Molecular mechanisms of RNA interference. Annu Rev Biophys.

[61] Li ML, Weng KF, Shih SR, Brewer G (2016). The evolving world of small RNAs from RNA viruses. Wiley Interdiscip Rev RNA.

[62] Zhang H, Kolb FA, Jaskiewicz L, Westhof E, Filipowicz W (2004). Single processing center models for human Dicer and bacterial RNase III. Cell.

[63] Ji X (2008). The mechanism of RNase III action: how dicer dices. Curr Top Microbiol Immunol.

[64] Kidwell MA, Chan JM, Doudna JA (2014). Evolutionarily conserved roles of the dicer helicase domain in regulating RNA interference processing. J Biol Chem.

[65] Svobodova E, Kubikova J, Svoboda P (2016). Production of small RNAs by mammalian Dicer. Pflugers Arch.

[66] Cerutti H, Casas-Mollano JA (2006). On the origin and functions of RNA-mediated silencing: from protists to man. Curr Genet.

[67] Mukherjee K, Campos H, Kolaczkowski B (2013). Evolution of animal and plant dicers: early parallel duplications and recurrent adaptation of antiviral RNA binding in plants. Mol Biol Evol.

[68] de Jong D, Eitel M, Jakob W, Osigus HJ, Hadrys H, Desalle R, Schierwater B (2009). Multiple dicer genes in the early-diverging metazoa. Mol Biol Evol.

[69] Shabalina SA, Koonin EV (2008). Origins and evolution of eukaryotic RNA interference. Trends Ecol Evol.

[70] Lau PW, Guiley KZ, De N, Potter CS, Carragher B, MacRae IJ (2012). The molecular architecture of human Dicer. Nat Struct Mol Biol.

[71] Machitani M, Sakurai F, Wakabayashi K, Tomita K, Tachibana M, Mizuguchi H (2016). Dicer functions as an antiviral system against human adenoviruses via cleavage of adenovirus-encoded noncoding RNA. Sci Rep.

[72] Birchler JA (2009). Ubiquitous RNA-dependent RNA polymerase and gene silencing. Genome Biol.

[73] Maida Y, Masutomi K (2011). RNA-dependent RNA polymerases in RNA silencing. Biol Chem.

[74] Spang A, Saw JH, Jorgensen SL, Zaremba-Niedzwiedzka K, Martijn J, Lind AE, van Eijk R, Schleper C, Guy L, Ettema TJ (2015). Complex archaea that bridge the gap between prokaryotes and eukaryotes. Nature.

[75] Koonin EV (2015). Archaeal ancestors of eukaryotes: not so elusive any more. BMC Biol.

[76] Koonin EV, Yutin N (2014). The dispersed archaeal eukaryome and the complex archaeal ancestor of eukaryotes. Cold Spring Harb Perspect Biol.

[77] MacRae IJ, Doudna JA (2007). Ribonuclease revisited: structural insights into ribonuclease III family enzymes. Curr Opin Struct Biol.

[78] Iyer LM, Koonin EV, Aravind L (2003). Evolutionary connection between the catalytic subunits of DNA-dependent RNA polymerases and eukaryotic RNA-dependent RNA polymerases and the origin of RNA polymerases. BMC Struct Biol.

[79] Salgado PS, Koivunen MR, Makeyev EV, Bamford DH, Stuart DI, Grimes JM (2006). The structure of an RNAi polymerase links RNA silencing and transcription. PLoS Biol.

[80] Qian X, Hamid FM, El Sahili A, Darwis DA, Wong YH, Bhushan S, Makeyev EV, Lescar J (2016). Functional Evolution in Orthologous Cell-encoded RNA-dependent RNA Polymerases. J Biol Chem.

[81] Buckley BA, Burkhart KB, Gu SG, Spracklin G, Kershner A, Fritz H, Kimble J, Fire A, Kennedy S (2012). A nuclear Argonaute promotes multigenerational epigenetic inheritance and germline immortality. Nature.

[82] Houri-Ze'evi L, Korem Y, Sheftel H, Faigenbloom L, Toker IA, Dagan Y, Awad L, Degani L, Alon U, Rechavi O (2016). A Tunable Mechanism Determines the Duration of the Transgenerational Small RNA Inheritance in C. elegans. Cell.

[83] Houri-Ze'evi L, Rechavi O. Plastic germline reprogramming of heritable small RNAs enables maintenance or erasure of epigenetic memories. RNA Biol. 2016;13(12):1212–17. 10.1080/15476286.2016.1229732 PMC5207387 27592591

[84] Iwasaki YW, Siomi MC, Siomi H (2015). PIWI-Interacting RNA: Its Biogenesis and Functions. Annu Rev Biochem.

[85] Czech B, Hannon GJ (2016). One Loop to Rule Them All: The Ping-Pong Cycle and piRNA-Guided Silencing. Trends Biochem Sci.

[86] Shalem O, Sanjana NE, Zhang F (2015). High-throughput functional genomics using CRISPR-Cas9. Nat Rev Genet.

[87] Barrangou R, Doudna JA (2016). Applications of CRISPR technologies in research and beyond. Nat Biotechnol.

[88] Koonin EV, Wolf YI (2016). Just how Lamarckian is CRISPR-Cas immunity: the continuum of evolvability mechanisms. Biol Direct.

[89] Leenay RT, Maksimchuk KR, Slotkowski RA, Agrawal RN, Gomaa AA, Briner AE, Barrangou R, Beisel CL (2016). Identifying and Visualizing Functional PAM Diversity across CRISPR-Cas Systems. Mol Cell.

[90] Hayes RP, Xiao Y, Ding F, van Erp PB, Rajashankar K, Bailey S, Wiedenheft B, Ke A (2016). Structural basis for promiscuous PAM recognition in type I-E Cascade from E. coli. Nature.

[91] Amitai G, Sorek R (2016). CRISPR-Cas adaptation: insights into the mechanism of action. Nat Rev Microbiol.

[92] Levy A, Goren MG, Yosef I, Auster O, Manor M, Amitai G, Edgar R, Qimron U, Sorek R (2015). CRISPR adaptation biases explain preference for acquisition of foreign DNA. Nature.

[93] Wei Y, Terns RM, Terns MP (2015). Cas9 function and host genome sampling in Type II-A CRISPR-Cas adaptation. Genes Dev.

[94] Makarova KS, Wolf YI, Koonin EV (2013). The basic building blocks and evolution of CRISPR-cas systems. Biochem Soc Trans.

[95] Nunez JK, Kranzusch PJ, Noeske J, Wright AV, Davies CW, Doudna JA (2014). Cas1-Cas2 complex formation mediates spacer acquisition during CRISPR-Cas adaptive immunity. Nat Struct Mol Biol.

[96] Nunez JK, Lee AS, Engelman A, Doudna JA. Integrase-mediated spacer acquisition during CRISPR-Cas adaptive immunity. Nature. 2015;519(7542):193–8. 10.1038/nature14237 PMC4359072 25707795

[97] Krupovic M, Koonin EV (2016). Self-synthesizing transposons: unexpected key players in the evolution of viruses and defense systems. Curr Opin Microbiol.

[98] Krupovic M, Makarova KS, Forterre P, Prangishvili D, Koonin EV (2014). Casposons: a new superfamily of self-synthesizing DNA transposons at the origin of prokaryotic CRISPR-Cas immunity. BMC Biol.

[99] Hickman AB, Dyda F (2015). The casposon-encoded Cas1 protein from Aciduliprofundum boonei is a DNA integrase that generates target site duplications. Nucleic Acids Res.

[100] Beguin P, Charpin N, Koonin EV, Forterre P, Krupovic M (2016). Casposon integration shows strong target site preference and recapitulates protospacer integration by CRISPR-Cas systems. Nucleic Acids Res.

[101] Koonin EV, Krupovic M (2015). Evolution of adaptive immunity from transposable elements combined with innate immune systems. Nat Rev Genet.

[102] Shmakov S, Abudayyeh OO, Makarova KS, Wolf YI, Gootenberg JS, Semenova E, Minakhin L, Joung J, Konermann S, Severinov K (2015). Discovery and Functional Characterization of Diverse Class 2 CRISPR-Cas Systems. Mol Cell.

[103] Shmakov S, Smargon A, Scott D, Cox D, Pyzocha N, Yan W, Abudayyeh OO, Gootenberg JS, Makarova KS, Wolf YI et al. Diversity and evolution of Class 2 CRISPR-Cas systems. Nature Rev Microbiol 2016, in press. 10.1038/nrmicro.2016.184 PMC5851899 28111461

[104] Pasternak C, Dulermo R, Ton-Hoang B, Debuchy R, Siguier P, Coste G, Chandler M, Sommer S (2013). ISDra2 transposition in Deinococcus radiodurans is downregulated by TnpB. Mol Microbiol.

[105] Dong D, Ren K, Qiu X, Zheng J, Guo M, Guan X, Liu H, Li N, Zhang B, Yang D (2016). The crystal structure of Cpf1 in complex with CRISPR RNA. Nature.

[106] Yamano T, Nishimasu H, Zetsche B, Hirano H, Slaymaker IM, Li Y, Fedorova I, Nakane T, Makarova KS, Koonin EV (2016). Crystal Structure of Cpf1 in Complex with Guide RNA and Target DNA. Cell.

[107] Kapitonov VV, Makarova KS, Koonin EV (2015). ISC, a Novel Group of Bacterial and Archaeal DNA Transposons That Encode Cas9 Homologs. J Bacteriol.

[108] Anantharaman V, Makarova KS, Burroughs AM, Koonin EV, Aravind L (2013). Comprehensive analysis of the HEPN superfamily: identification of novel roles in intra-genomic conflicts, defense, pathogenesis and RNA processing. Biol Direct.

[109] Abudayyeh OO, Gootenberg JS, Konermann S, Joung J, Slaymaker IM, Cox DB, Shmakov S, Makarova KS, Semenova E, Minakhin L (2016). C2c2 is a single-component programmable RNA-guided RNA-targeting CRISPR effector. Science.

[110] Kapitonov VV, Koonin EV (2015). Evolution of the RAG1-RAG2 locus: both proteins came from the same transposon. Biol Direct.

[111] Kaya E, Doxzen KW, Knoll KR, Wilson RC, Strutt SC, Kranzusch PJ, Doudna JA (2016). A bacterial Argonaute with noncanonical guide RNA specificity. Proc Natl Acad Sci U S A.

[112] Swarts DC, Koehorst JJ, Westra ER, Schaap PJ, van der Oost J (2015). Effects of Argonaute on Gene Expression in Thermus thermophilus. PLoS ONE.

[113] Martin W, Hoffmeister M, Rotte C, Henze K (2001). An overview of endosymbiotic models for the origins of eukaryotes, their ATP-producing organelles (mitochondria and hydrogenosomes), and their heterotrophic lifestyle. Biol Chem.

[114] Martin W, Koonin EV (2006). Introns and the origin of nucleus-cytosol compartmentation. Nature.

[115] Embley TM, Martin W (2006). Eukaryotic evolution, changes and challenges. Nature.

[116] Koonin EV (2006). The origin of introns and their role in eukaryogenesis: a compromise solution to the introns-early versus introns-late debate?. Biol Direct.

[117] Koonin EV, Dolja VV, Krupovic M (2015). Origins and evolution of viruses of eukaryotes: The ultimate modularity. Virology.

[118] Makarova KS, Wolf YI, Koonin EV (2013). Comparative genomics of defense systems in archaea and bacteria. Nucleic Acids Res.

[119] Kobayashi I (2001). Behavior of restriction-modification systems as selfish mobile elements and their impact on genome evolution. Nucleic Acids Res.

[120] Van Melderen L, Saavedra De Bast M (2009). Bacterial toxin-antitoxin systems: more than selfish entities?. PLoS Genet.

[121] He F, Chen L, Peng X (2014). First experimental evidence for the presence of a CRISPR toxin in sulfolobus. J Mol Biol.

[122] Koonin EV, Makarova KS (2013). CRISPR-Cas: evolution of an RNA-based adaptive immunity system in prokaryotes. RNA Biol.

[123] Makarova KS, Anantharaman V, Aravind L, Koonin EV (2012). Live virus-free or die: coupling of antivirus immunity and programmed suicide or dormancy in prokaryotes. Biol Direct.

[124] Koonin EV, Zhang F (2017). Coupling immunity and programmed cell suicide in prokaryotes: Life-or-death choices. Bioessays.

[125] Koonin EV (2014). The double-edged sword of Lamarck: comment on “Diversity, evolution, and therapeutic applications of small RNAs in prokaryotic and eukaryotic immune systems” by Edwin L. Cooper and Nicola Overstreet. Phys Life Rev.

[126] Roche B, Arcangioli B, Martienssen RA. RNA interference is essential for cellular quiescence. Science. 2016;354(6313). 10.1126/science.aah5651 PMC5858868 27738016

[127] Westra ER, Buckling A, Fineran PC (2014). CRISPR-Cas systems: beyond adaptive immunity. Nat Rev Microbiol.

[128] Nowacki M, Shetty K, Landweber LF (2011). RNA-Mediated Epigenetic Programming of Genome Rearrangements. Annu Rev Genomics Hum Genet.

[129] Swart EC, Nowacki M (2015). The eukaryotic way to defend and edit genomes by sRNA-targeted DNA deletion. Ann N Y Acad Sci.

[130] Wang J, Davis RE (2014). Programmed DNA elimination in multicellular organisms. Curr Opin Genet Dev.

[131] Storz G, Vogel J, Wassarman KM (2011). Regulation by small RNAs in bacteria: expanding frontiers. Mol Cell.

[132] Updegrove TB, Zhang A, Storz G (2016). Hfq: the flexible RNA matchmaker. Curr Opin Microbiol.

[133] Lapidot M, Pilpel Y (2006). Genome-wide natural antisense transcription: coupling its regulation to its different regulatory mechanisms. EMBO Rep.

[134] Yuan C, Wang J, Harrison AP, Meng X, Chen D, Chen M (2015). Genome-wide view of natural antisense transcripts in Arabidopsis thaliana. DNA Res.

[135] Patel BH, Percivalle C, Ritson DJ, Duffy CD, Sutherland JD (2015). Common origins of RNA, protein and lipid precursors in a cyanosulfidic protometabolism. Nat Chem.

[136] Hlevnjak M, Polyansky AA, Zagrovic B (2012). Sequence signatures of direct complementarity between mRNAs and cognate proteins on multiple levels. Nucleic Acids Res.

[137] Wolf YI, Koonin EV (2007). On the origin of the translation system and the genetic code in the RNA world by means of natural selection, exaptation, and subfunctionalization. Biol Direct.

